# A Magnetically Controlled Soft Microrobot Steering a Guidewire in a Three-Dimensional Phantom Vascular Network

**DOI:** 10.1089/soro.2018.0019

**Published:** 2019-02-11

**Authors:** Sungwoong Jeon, Ali Kafash Hoshiar, Kangho Kim, Seungmin Lee, Eunhee Kim, Sunkey Lee, Jin-young Kim, Bradley J. Nelson, Hyo-Jeong Cha, Byung-Ju Yi, Hongsoo Choi

**Affiliations:** ^1^ Department of Robotics Engineering, Daegu Gyeongbuk Institute of Science and Technology (DGIST), Daegu, South Korea.; ^2^ DGIST-ETH Microrobotics Research Center (DEMRC), DGIST, Daegu, South Korea.; ^3^ Institute of Robotics and Intelligent Systems, ETH Zurich, Zurich, Switzerland.; ^4^ Department of Electronic Systems Engineering, Hanyang University, Ansan, Korea.

**Keywords:** soft microrobot, guidewire, percutaneous coronary intervention (PCI), steerability, intravascular treatments, magnetic steering

## Abstract

Magnetically actuated soft robots may improve the treatment of disseminated intravascular coagulation. Significant progress has been made in the development of soft robotic systems that steer catheters. A more challenging task, however, is the development of systems that steer sub-millimeter-diameter guidewires during intravascular treatments; a novel microrobotic approach is required for steering. In this article, we develop a novel, magnetically actuated, soft microrobotic system, increasing the steerability of a conventional guidewire. The soft microrobot is attached to the tip of the guidewire, and it is magnetically steered by changing the direction and intensity of an external magnetic field. The microrobot is fabricated via replica molding and features a soft body made of polydimethylsiloxane, two permanent magnets, and a microspring. We developed a mathematical model mapping deformation of the soft microrobot using a feed-forward approach toward steering. Then, we used the model to steer a guidewire. The angulation of the microrobot can be controlled from 21.1° to 132.7° by using a magnetic field of an intensity of 15 mT. Steerability was confirmed by two-dimensional *in vitro* tracking. Finally, a guidewire with the soft microrobot was tested by using a three-dimensional (3D) phantom of the coronary artery to verify steerability in 3D space.

## Introduction

Over the past few years, untethered microrobots have found diverse applications in biopsy,^1,2^ treatment of hyperthermia,^3^ cell culture,^4,5^ targeted drug delivery,^6–8^ tissue scaffolding,^9^ sensing,^10^ tissue marking,^11^ and microsurgery.^12–14^ More recently, soft micromanipulators have been used for targeted drug delivery.^15^ The soft deformable structures of such microrobots render them well suited to perform biomedical operations within vascular networks.

Cardiovascular diseases were responsible for one of every three deaths worldwide in 2013, and chronic total occlusions are found in about one-third of patients with coronary artery disease.^16,17^ Percutaneous coronary intervention (PCI) (a minimally invasive procedure) can be used to unblock coronary arteries.^18–20^ PCI features the use of guidewires and catheters. PCI is performed by inserting a catheter into the femoral or radial artery and steering that catheter into the heart. The process is monitored by using an X-ray imaging system (angiography). The catheter is positioned inside the coronary artery, and the X-ray image is used to map the arteries and locate the blockage or narrowing. Then, a guidewire is inserted into the artery and steered to the target position. If necessary, a balloon catheter can be delivered to the target position and the artery is inflated. During this procedure, conventional guidewires are manually controlled by back-and-forth pushing and rotating. This prolongs the operation time and increases the radiation exposure of both patients and doctors. Further, the procedural success rate is highly dependent on the skill and experience of the physician. Thus, a number of research groups have attempted to develop steerable guidewire systems, as discussed later.

The use of elastic tubes has been studied in this context.^21^ However, contact with vessel walls and the associated friction, which compromise precision, have imposed limits on their application. Shape memory alloy actuators <2 mm in diameter have been used to steer catheters.^22^ However, the maximal bending angle was <90°, and the need for submillimeter design remained unanswered. More recently, soft actuators made of various materials have been developed to steer catheters.^23^ Eight deformations were programmed by using a microcontroller, and a 10-g load was used to evaluate performance. Further, a scaled version of a three-dimensional (3D)-printed carotid artery was utilized for steering. Despite successful proof-of-concept, temperature and size limitations remain. Magnetically controlled catheters have attracted significant attention over the past few years because of their precise and responsive steerability.

Magnetic resonance imaging (MRI)-based actuation has been used to remotely steer and monitor catheters,^24^ and the relationship between magnetic torque and the mechanical restoration of torque has been modeled.^25^ However, the model employs linear terms for simplicity and, thus, fails to predict nonlinear deformations. Further, the MRI-based systems are not tailored to endovascular interventions. Magnetic actuation systems for steering catheters have been proposed,^26,27^ as have position control schemes.^28,29^ However, guidewire steering is needed for many intravascular applications, such as PCI.

In a previous study, a magnet was attached to the tip of a guidewire and two permanent magnets positioned at either side of a fluoroscopy table were used to steer the guidewire.^30^ Although the guidewire became aligned with the direction of the field, the use of permanent magnets for steering limits the ability to control the magnetic field. MRI-based guidewire steering has also been studied, but image reconstruction is time-consuming and the need for sequential imaging/steering compromises the utility of the approach.^31,32^ Therefore, we developed a soft submillimeter-diameter microrobot mounted at the end of a conventional guidewire. The microrobot is steered by using a dedicated actuation system consisting of eight electromagnets.

We used polydimethylsiloxane (PDMS, Sylgard 184; Dow Corning Corp., Midland, MI), which has a low elastic modulus and a high Poisson ratio, to fabricate the soft microrobot that incorporates two permanent magnets, allowing steering by an external magnetic field within a phantom vascular network. We developed a mathematical model of the relationship between the magnetic force and microrobot deformation. As this was nonlinear, the force-deformation relationship was modeled via finite element analysis (FEA). Deformation angles of up to 132.7° were experimentally achieved. Two-dimensional (2D) and 3D tracking experiments showed that steering and tracking performance was reliable.

## Materials and Methods

### The soft microrobot

**FIG. 1.**
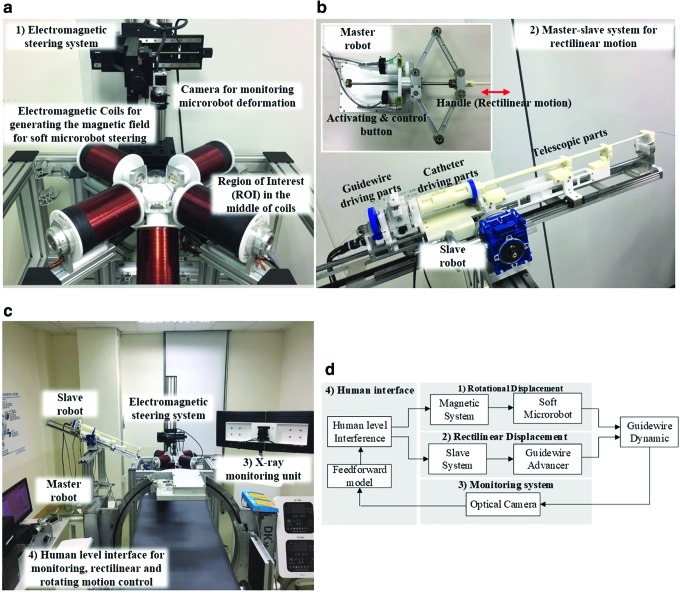
Overview of the magnetically actuated microrobotic system. **(a)** The OctoMag actuation system for microrobot steering; **(b)** the master/slave robotic system controlling rectilinear motion; **(c)** the X-ray monitoring system (not used in the present work) and the user interface; and **(d)** the schematic and the connections for guidewire steering.

The steering system has four major components (Fig. 1). A magnetic field steers the soft microrobot. Figure 1a shows the magnetic actuation system (OctoMag; Aeon Scientific GmbH, Schlieren, Switzerland), which consists of eight electromagnets generating a 3D magnetic field of a maximum intensity of 40 mT (for details see Kummer *et al.*^12^). Guidewire rectilinear motion is created by using a master/slave system (Fig. 1b). The master is activated by the operator, and the slave generates rectilinear motion. The system can be used to remotely control commercial guidewires and catheters intravascularly (for details Cha *et al.*^33^). Once the microrobot points in the desired direction, the slave moves forward and advances the guidewire and the microrobot.

The complete system, shown in Figure 1c, features an X-ray system enabling real-time monitoring similar to that of conventional PCI. In this study, however, optical monitoring was employed for convenience; X-ray monitoring will be studied in future works.

Figure 1d shows an overview of the system and its connections. Rotational and rectilinear displacements are triggered via magnetic actuation and the master/slave system, respectively. Feedback is provided by a camera. We developed a model of the rectilinear and angular displacements, and a user interface to control both processes.

### The magnetic steering system

**FIG. 2.**
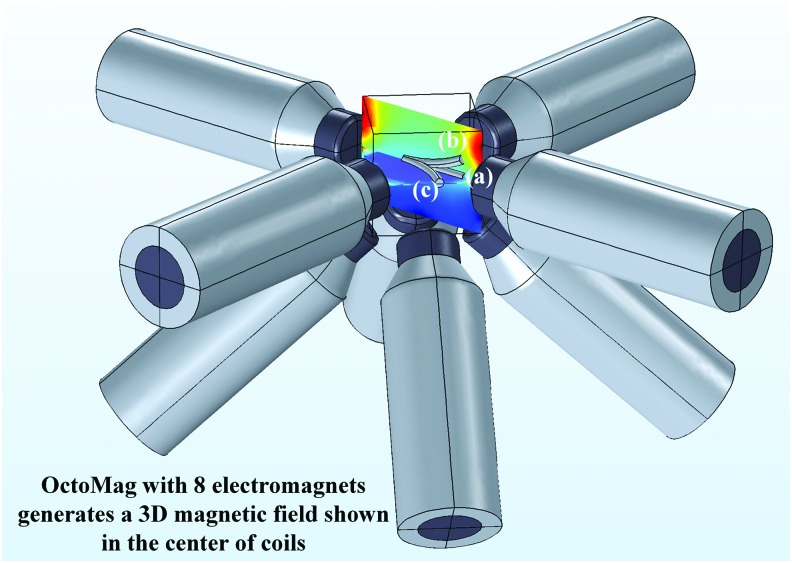
A schematic of the OctoMag system^12^ and the soft microrobot. The microrobot is steered by changes in the direction of the magnetic field. The microrobot dimensions are exaggerated to afford better visualization; the microrobot is illustrated in three different positions: (a) the initial position; (b) after deformation in the *z* direction; and (c) after deformation in the *x* direction.

The magnetic steering system consists of the OctoMag system and the novel soft microrobot (Fig. 2). Two permanent magnets were placed at equal distance within the PDMS matrix and used to steer the guidewire. The OctoMag actuation system^12^ can generate a 3D magnetic field of constant magnitude that varies in terms of direction. As the field direction changes, the microrobot experiences a magnetic torque, forcing realignment in the direction of the field. Thus, the microrobot is magnetically steered in the direction of interest (Fig. 2).

## Model of Magnetic Actuation

### Mathematical model

The magnetic field in current-free space is described by Maxwell's equations as:

\begin{align*}
\nabla \cdot \vec { B} = 0 \tag{1}
\end{align*}

\begin{align*}
\nabla \times \vec { B} = 0 \tag{2}
\end{align*}

where $\vec B$ is the $3 \times 1$ magnetic field vector. Considering the boundary conditions, these equations describe the magnetic field. The force and torque on a magnetic object in this magnetic field are given by the following equations:

\begin{align*}
\vec F = \left( { \vec m \cdot \nabla } \right) \vec B \tag{3}
\end{align*}

\begin{align*}
\vec \tau = \vec m \times \vec B \tag{4}
\end{align*}

where $\vec F$ and $\vec \tau$ are $3 \times 1$ force and torque vectors, respectively, and $\vec m$ is the $3 \times 1$ dipole moment of the magnetic object. Maxwell's equations impose the following two constraints: (1) Equation (1) indicates that the gradient matrix has a zero trace, and (2) Equation (2) shows that the gradient matrix is symmetrical. Therefore, Equation (3) can be written as:

\begin{align*}
\vec F = \left[ { \begin{matrix} { { f_x } } \\ { { f_y } } \\
{ { f_z } } \\ \end{matrix} } \right] = \left[ { \begin{matrix} {
{ \frac { \partial { { \rm B } _x } }  { \partial x } } } & { {
\frac { \partial { { \rm B } _x } }  { \partial y } } } & { {
\frac { \partial { { \rm B } _x } }  { \partial z } } } \\ { {
\frac { \partial { { \rm B } _x } }  { \partial y } } } & { {
\frac { \partial { { \rm B } _y } }  { \partial y } } } & { {
\frac { \partial { { \rm B } _y } }  { \partial x } } } \\ { {
\frac { \partial { { \rm B } _x } }  { \partial z } } } & { {
\frac { \partial { { \rm B } _y } }  { \partial z } } } & { - ( {
\frac { \partial { { \rm B } _y } }  { \partial z } } + { \frac {
\partial { { \rm B } _x } }  { \partial x } } ) } \\ \end{matrix} } \right] \left[ { \begin{matrix} { { m_x } } \\ { { m_y } }
\\ { { m_z } } \\ \end{matrix} } \right] \tag { 5 }
\end{align*}

The magnetic field gradient represented by Equation (5) has five components, which can be represented as a $5 \times 1$ matrix (*B_g_*). Consequently, Equation (5) can be rearranged as:

\begin{align*}
\vec F = \left[ {\begin{matrix} { { f_x } } \\
{ { f_y } } \\ { { f_z } } \\ \end{matrix} } \right] = & \left[
{\begin{matrix} { { m_x } } & { { m_y } } & { { m_z } } & 0 & 0
\\ 0 & { { m_x } } & 0 & { { m_y } } & { { m_z } } \\ { - { m_z }
} & 0 & { { m_x } } & { - { m_z } } & { { m_y } } \\ \end{matrix}
} \right] \left[ \begin{matrix} { \frac { \partial { B_x } } { dx
} } \hfill \\ { \frac { \partial { B_x } }  { dy } } \hfill
\\ { \frac { \partial { B_x } }  { dz } } \hfill \\ { \frac {
\partial { B_y } }  { dy } } \hfill \\ { \frac { \partial { B_y }
}  { dz } } \hfill \\ \end{matrix} \right] \\ = & { M_F } { \kern
1pt } { B_g } \\  \tag { 6 }
\end{align*}

where *M_F_* packs the dipole terms contributing to the magnetic force. Considering the cross-product in Equation (4), the magnetic torque vector can be represented as:

\begin{align*}
\begin{split} \vec \tau = \left[ \begin{matrix} { \tau _x} \hfill \\ { \tau _y} \hfill \\ { \tau _z} \hfill \\\end{matrix}  \right] = & \left[ { \begin{matrix} 0 & { - m} & {{m_y}} \\ {{m_z}} & 0 & { - {m_x}} \\ { - {m_y}} & {{m_x}} & 0 \\ \end{matrix} } \right] \left[ \begin{matrix} {B_x} \hfill \\ {B_y} \hfill \\ {B_z} \hfill \\\end{matrix}  \right] \\ = & {M_ \tau }{ \kern 1pt} B \\\end{split}
  \tag{7}
\end{align*}

where ${M_ \tau }$ packs the dipole terms contributing to magnetic torque. Combining Equations (6) and (7), the total force and torque matrix can be written as:

\begin{align*}
{ \left[ \begin{matrix} \tau \hfill \\ F \hfill \\\end{matrix}  \right] _{6 \times 1}} = { \left[ { \begin{matrix} {{M_ \tau }} & 0 \\ 0 & {{M_F}} \\ \end{matrix} } \right] _{6 \times 8}}{ \left[ \begin{matrix} B \hfill \\ {B_g} \hfill \\\end{matrix}  \right] _{8 \times 1}} \tag{8}
\end{align*}

The system outputs (torque and force) depend on this $6 \times 1$ matrix. When investigating the soft microrobot system, the microrobot structure was considered to be isotropic in the x and z directions with two degrees of freedom (DOFs) (${D_{yx}}$ deformation in the yx plane in the x direction, and ${D_{yz}}$ deformation in the yz plane in the z direction). The deformations can be written as:

\begin{align*}
{ \left[ {{D_{yz}}} \right] _{3 \times 1}} = \sum \limits_{i = 1}^n {{ \beta _i}} { \vec \tau _{ix}} = \sum \limits_{i - 1}^n {{ \alpha _i}} \,{ \vec f_{iz}} \tag{9}
\end{align*}

\begin{align*}
{ \left[ {{D_{xz}}} \right] _{3 \times 1}} = \sum \limits_{i = 1}^n {{ \beta _i}} { \vec \tau _{iz}} = \sum \limits_{i - 1}^n {{ \alpha _i}} \,{ \vec f_{ix}} \tag{10}
\end{align*}

The $\alpha \;$and$\; \beta$ terms reflect the mechanical properties (elasticity, shear modulus) and structural geometry that map the forces and torques to the deformations, and *n* is the number of permanent magnets in the microrobot. Now, considering Equation (9), and using Equation (8), the relationship between the magnetic field and deformation can be written as:

\begin{align*}
\begin{split} & { \left[ {{D_{yz}}} \right] _{3 \times 1}} = { \sum \limits_{i = 1}^n { \left[ { \begin{matrix} 0 & { - {m_{zi}}} & {{m_{yi}}} & {{m_{xi}}} & {{m_{yi}}} & {{m_{zi}}} & 0 & 0 \\ {{m_{zi}}} & 0 & { - {m_{xi}}} & 0 & {{m_{xi}}} & 0 & {{m_{yi}}} & {{m_{zi}}} \\ { - {m_{yi}}} & {{m_{xi}}} & 0 & { - {m_{zi}}} & 0 & {{m_{xi}}} & { - {m_{zi}}} & {{m_{yi}}} \\ \end{matrix} } \right] } _{3 \times 8}}{ \left[ K \right] _{8 \times 8}}{ \left[ \begin{matrix} B \hfill \\ {B_g} \hfill \\\end{matrix}  \right] _{8 \times 1}} \\ & {K_{8 \times 8}} = diag ( { \beta _i} , { \beta _i} , { \beta _i} , { \alpha _i} , { \alpha _i} , { \alpha _i} , { \alpha _i} , { \alpha _i} ) \\\end{split}
  \tag{11}
\end{align*}

**FIG. 3.**
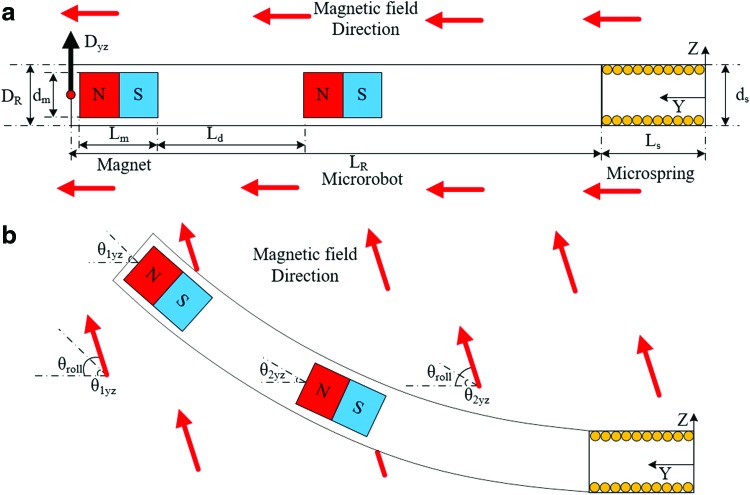
Magnetic actuation of the microrobot to steer the guidewire. **(a)** The geometrical parameters of the microrobot in the initial position: *D_R_*, microrobot diameter, *d_m_*, magnet diameter, *L_R_*, microrobot length, *L_m_*, magnet length, and *L_d_*, the distance between the magnets. **(b)** Microrobot deformation with a change in the magnetic field, *θ_roll_* is the field direction, and *θ_1_* and *θ_2_* are the deformation angles of the first and second magnets, respectively. The angular differences between the magnets and the field are represented by *θ_1yz_* and *θ_2yz_*.

Figure 3 shows the geometry of the soft microrobot and the configuration used for steering. The magnetization vector depends on the orientation of the permanent magnets, which was initially chosen to be the y direction; thus: ${m_1} = {m_2} = m \left[ {0 \;1 \;0} \right]$. However, the direction of the magnetization vector changes as the position of the microrobot changes, as shown in Figure 3. The orientations of the magnets are represented by ${ \theta _{1yz}}$, ${ \theta _{2yz}}$, ${ \theta _{1yx}}$, and ${ \theta _{2yx}}$ (Fig. 3). Therefore, considering both the rotational angles and the directions of initial magnetization, the magnetization vector for each magnet *i* can be written as:

\begin{align*}
\left[ \begin{matrix} {m_{xi}} \hfill \\ {m_{yi}} \hfill \\ {m_{zi}} \hfill \\\end{matrix}  \right] = \left[ { \begin{matrix} {c{ \theta _{iyx}}} & { - s{ \theta _{iyx}}} & 0 \\ {c{ \theta _{iyz}}\,s{ \theta _{iyx}}} & {c{ \theta _{iyz}}\,c{ \theta _{iyx}}} & { - s{ \theta _{iyz}}} \\ {s{ \theta _{iyz}}\,s{ \theta _{iyx}}} & {s{ \theta _{iyz}}\,c{ \theta _{iyx}}} & {c{ \theta _{iyz}}} \\ \end{matrix} } \right] \left[ \begin{matrix} 0 \hfill \\ m \hfill \\ 0 \hfill \\\end{matrix}  \right] \tag{12}
\end{align*}

where *s* and *c* indicate *sin* and *cos*, respectively. When steering the soft microrobot, the magnetic gradient is considered to be zero and magnetic torque (only) is used for steering. Consequently, considering Equation (12), Equation (11) can be written as:

\begin{align*}
\begin{split} & { \left[ {{D_{yz}}} \right] _{3 \times 1}} \sum \limits_{i = 1}^n { \left[ { \begin{matrix} 0 & { - m ( s{ \theta _{iyz}}c{ \theta _{iyx}} ) } & { - m ( c{ \theta _{iyz}}c{ \theta _{iyx}} ) } \\ { - m ( s{ \theta _{iyz}}c{ \theta _{iyx}} ) } & 0 & { - m ( s{ \theta _{iyx}} ) } \\ { - m ( c{ \theta _{iyz}}c{ \theta _{iyx}} ) } & { - m ( s{ \theta _{iyx}} ) } & 0 \\ \end{matrix} } \right] } _{3 \times 3}^{}diag ( { \beta _i} , { \beta _i} , { \beta _i} ) \left[ { \begin{matrix} {{ \beta _x}} \\ {{ \beta _y}} \\ {{ \beta _z}} \\ \end{matrix} } \right] \\ & { \left[ D \right] _{3 \times 1}} \left( { \sum \limits_{i = 1}^n {{{ \left[ {{M_i}} \right] }_{3 \times 3}}{{ \left[ {{ \beta _i}} \right] }_{3 \times 3}}} } \right) { \left[ B \right] _{3 \times 1}} \\\end{split}
  \tag{13}
\end{align*}

In Equation (13), the matrix *M_i_* is the magnetization vector for each magnet, ${ \beta _i}$ maps the magnetic torque to the deformation, and the matrix *B* is the magnetic field. To steer the soft microrobot, we consider the field to be constant in the y direction [0 B 0]^T^ and change the field direction to generate the magnetic torque according to Equation (13). Therefore, deformation can be described as:

\begin{align*}
{ \left[ D \right] _{3 \times 1}} \left( { \sum \limits_{i = 1}^n {{{ \left[ {{M_i}} \right] }_{3 \times 3}}{{ \left[ {{B_i}} \right] }_{3 \times 3}}} } \right) \left[ { \begin{matrix} 1 & 0 & 0 \\ 0 & {c{ \theta _{roll}}} & { - s{ \theta _{roll}}} \\ 0 & {s{ \theta _{roll}}} & {c{ \theta _{roll}}} \\ \end{matrix} } \right] \left[ { \begin{matrix} {c{ \theta _{yaw}}} & { - s{ \theta _{yaw}}} & 0 \\ {s{ \theta _{yaw}}} & {c{ \theta _{yaw}}} & 0 \\ 0 & 0 & 1 \\ \end{matrix} } \right] \left[ { \begin{matrix} 0 \\ B \\ 0 \\ \end{matrix} } \right] \tag{14}
\end{align*}

where *s* and *c* indicate *sin* and *cos*, respectively, *B* is the constant magnetic field, and ${ \theta _{roll}}$ and$\;{ \theta _{yaw}}$ are the roll and yaw angles about the *x* and *z* axes, respectively. Finally, Equation (14) can be packed as:

\begin{align*}
{ \left[ D \right] _{3 \times 1}} \left( { \sum \limits_{i = 1}^n { \left[ {{{ \left[ {{M_i}} \right] }_{3 \times 3}}{{ \left[ {{B_i}} \right] }_{3 \times 3}}} \right] } } \right) { \left[ {{R_{roll}}} \right] _{3 \times 3}}{ \left[ {{R_{yaw}}} \right] _{3 \times 3}}{ \left[ B \right] _{3 \times 1}}  \tag{15}
\end{align*}

where ${R_{roll}}$ and ${R_{yaw}}$ are the rotational matrices about the *x* and *z* axes, *B* is the constant magnetic field in the *y* direction, the matrix *M_i_* is the magnetization vector for each magnet, and ${ \beta _i}$ maps the magnetic torque to the deformations. Equation (15) thus yields the deformation. However, the matrix *M* is a function of the magnitude of magnetization (*m*) and the magnet configurations (${ \theta _{1yz}}$, ${ \theta _{2yz}}$, ${ \theta _{1yx}}$, and ${ \theta _{2yx}}$); and ${ \beta _i}$ depends on the mechanical properties and structural geometry of the microrobot. Structural nonlinearity limits the mathematical modeling; thus, FEA was used to determine the deformations.

### The FEA model for the soft microrobot

To simplify the model, the soft microrobot was assumed to be isotropic and the model was derived in 2D. Deformation of the soft microrobot by an external magnetic field was studied by using the COMSOL Multiphysics^®^ suite v. 5.3. (COMSOL AB, Stockholm, Sweden). The microrobot geometry is shown in Figure 3, and the detailed geometrical parameters are listed in Table 1. As shown in Figure 3, the soft microrobot consists of two permanent magnets and a PDMS cantilever beam. The material properties and simulation conditions are given in Tables 2 and 3, respectively. The microrobot was modeled by using the geometrical information in Table 1, and a triangular element was used to generate the mesh.

**Table 1.** Geometrical Information Used in the Fabrication and Finite Element Analysis Simulation of the Soft Microrobot

	*Diameter*		*Length*	
	*Parameter*	*Value (μm)*	*Parameter*	*Value (μm)*
Microrobot	D_R_	500	L_R_	3,800
Magnet	d_m_	400	L_m_	800
Microspring	d_s_	500	L_s_	2000
Guidewire	d_g_	360	L_g_	1.9 × 10^6^

**Table 2.** Material Properties of the Soft Microrobot Used in Mathematical Modeling and Finite Element Analysis^34,42,43^

*Material*	*Polydimethylsiloxane*	*Neodymium magnet (NdFeB, N52)*
Density ($kg / {m^3}$)	0.97	7,500
Young's modulus (Pa)	600 × 10^3^	160 × 10^9^
Poisson ratio	0.49	0.24
Relative permeability	1	1.05
Remanence flux density (T)	_	1.43

**Table 3.** Experimental and Simulation Conditions for Steering Mediated by the Soft Microrobot

*Parameter*	*Value*
Direction of the magnetic field (°)	From 30 to 170
Intensity of the magnetic field (mT)	5, 10, 15

**FIG. 4.**
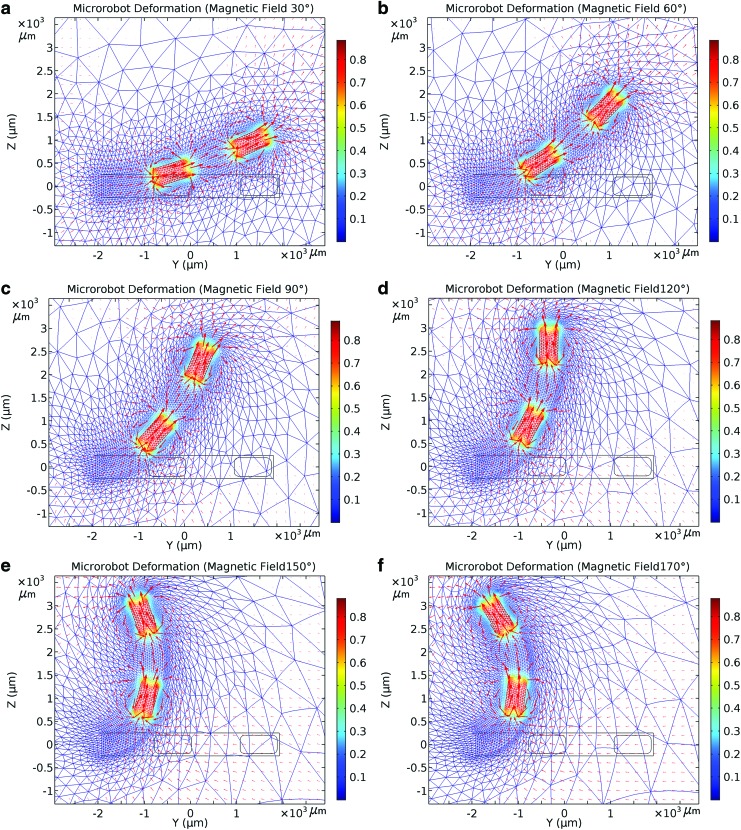
Finite element analysis simulation in the COMSOL multiphysics environment. Deformations of the microrobot by an external magnetic field of intensity of 15 mT at angles of **(a)** 30°, **(b)** 60°, **(c)** 90°, **(d)** 120°, **(e)** 150°, and **(f)** 170°. The *right color bar* shows the magnetic flux density (T).

The external field magnitude was considered to be a maximum of 15 mT, and the field direction was initially designed to align in the *y* direction. The magnetic field direction (*θ_roll_*) was varied between 10° and 170°, and structural analysis was used to determine deformation with respect to the change in that direction. Figure 4 shows the deformations associated with various directions of the magnetic field. The results are presented and discussed in the Results section.

## Fabrication of the soft microrobot

The microrobot must be of submillimeter scale, exhibit high deformability and steerability. The micrometer scale enables the guidewire with the soft microrobot (GSM) to be inserted into coronary arteries. High-level deformability enables guidance over a wide range of branch angles, and high steerability affords precise control. Therefore, we fabricated the microrobot from PDMS and incorporated two permanent magnets (NdFeB, N52; Ningbo Zhonghang Magnetic Materials Co., Ltd, Zhejiang, China). The microrobot is cylindrical in shape (thus symmetrical), affording multi-axis steerability. The microrobot geometry and material properties are listed in Tables 1 and 2, respectively.

**FIG. 5.**
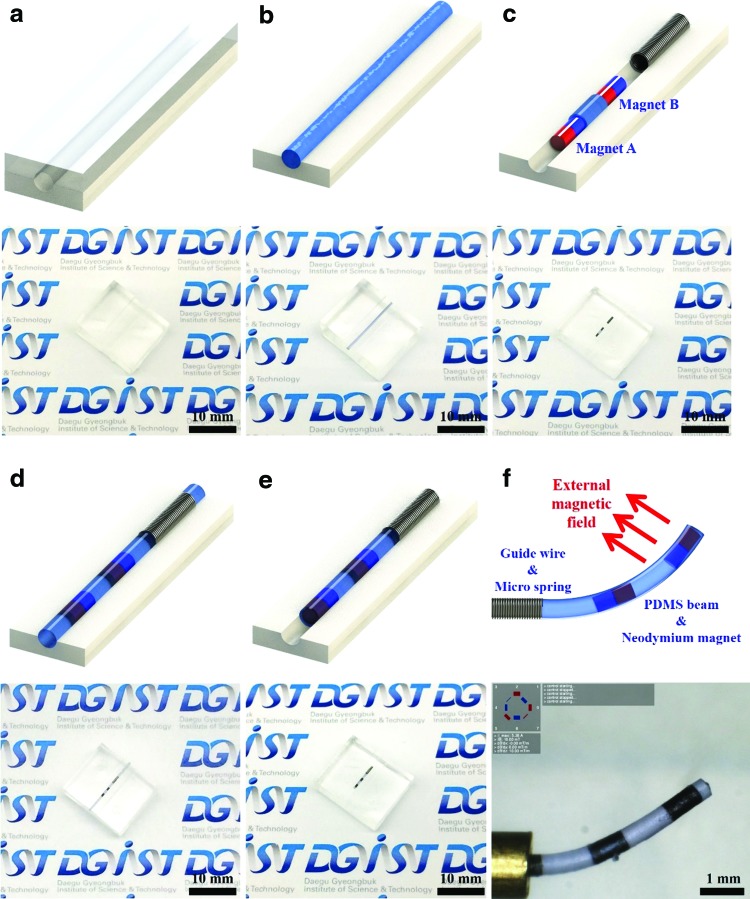
Fabrication of the soft microrobot composed of a beam (polydimethylsiloxane [PDMS] silicone and a neodymium magnet), a connection (a microspring), and a guidewire. **(a)** The PDMS metal master mold and replica molding using PDMS; **(b)** pouring of the silicone elastomer mixture into the metal PDMS master mold, and curing; **(c)** alignment of the two-magnet, 1-mm-diameter rod-shaped PDMS block and the microspring; **(d)** pouring of the silicone elastomer mixture into the PDMS master mold, and curing; **(e)** adjusting the length of the microrobot; and **(f)** connecting the microrobot to the guidewire to allow guidewire steering.

A metal master, commonly used for replica molding, served as the master for PDMS molding.^34,35^ The metal master was made from stainless steel and was manufactured by wire electrical discharge machining; thus, it can be reused multiple times. We first prepared a replica PDMS mold (a PDMS master) by using a metal master. A mixture of PDMS prepolymer and the curing agent was poured into the metal master, and the replica PDMS mold was obtained by peeling the metal master away. The next step was re-molding by using a PDMS master; this second PDMS mold was used to fabricate the soft microrobot. PDMS is optically transparent, which aids fabrication (as shown in Fig. 5). As the mold and beam of the microrobot are made of the same material (PDMS), they tend to stick together after the beam is cured. We, thus, hydrophobically coated the mold and the beam by using an oxygen plasma system (Cute; FEMTO SCIENCE, Seoul, Korea) and trichloro(1H,1H,2H,2H-perfluorooclyl)silane (Sigma-Aldrich, St. Louis, MO). Thin layers of perfluorooctyl trichlorosilane were deposited on the PDMS surfaces in a closed chamber under a pressure of 0.5 bar at 80°C for 2 h.^36^ This improved detachment of the beam from the mold.

The fabrication process is shown in Figure 5. Initially, the PDMS mold was filled with a Sylgard 184 silicone elastomer mixture (Dow Corning Corp., Midland, MI) at a base:curing agent weight ratio of 10:1 by using methyl blue to visualize the elastomer mixture inside the mold, and the mixture was then cured at 80°C for 8 h^36^ (Fig. 5b). A rod-shaped PDMS beam (1 mm) was used in the next fabrication step. The beam, permanent magnets, and microspring were aligned on the mold, as shown in Figure 5c. The microspring was used to connect the PDMS beam to a guidewire. The distance between the magnet B and the microspring was 1 mm. As shown in Figure 5d, the silicone elastomer mixture was then used to fill the mold in the closed chamber under vacuum followed by curing at 80°C for 8 h. The final structure is shown in Figure 5e. Finally, the microrobot was connected to a conventional guidewire (Fig. 5f). In this approach, a conventional commercially available guidewire was used for navigation. This guidewire is in clinical use, so its application does not raise any toxicity issues. The permanent magnets in the microrobot are enclosed within biocompatible PDMS material. The biocompatibility test results for the PDMS material revealed no toxicity (Supplementary Fig. S2). Therefore, the microrobot is safe for future use in *in-vivo* studies.^37^

### Experimental setup

**FIG. 6.**
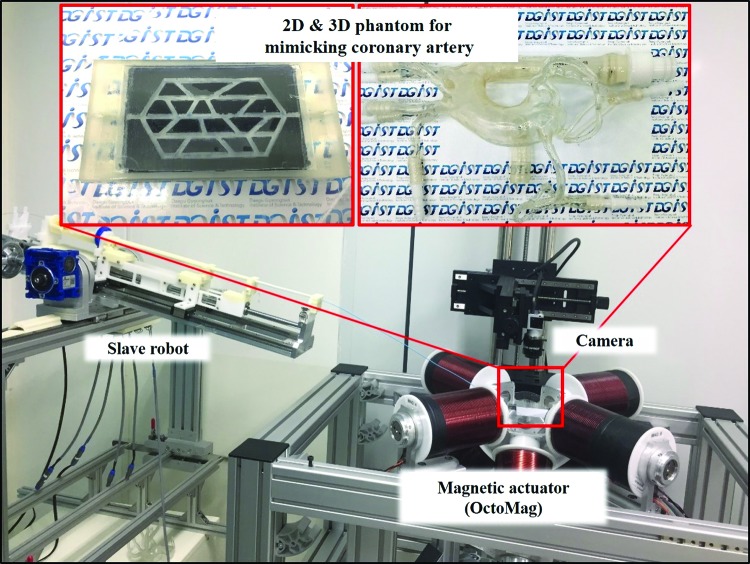
The magnetic actuator (OctoMag), master/slave system, two-dimensional (2D) and three-dimensional (3D) blood vessel phantoms, and the charge-coupled device camera. The soft microrobot steered the guidewire in 2D and 3D experiments.

The OctoMag system precisely changes the direction of the magnetic field.^12^ Rectilinear motion of the GSM was controlled by a master/slave system^33^ (Fig. 6). The OctoMag system features eight Helmholtz/Maxwell electromagnetic coils that are capable of generating a maximum magnetic field of 120 mT in a region of interest (a sphere 8 cm in radius). The OctoMag system afforded five DOFs to untethered microrobots (three position DOFs, two orientation DOFs).^38–40^ The system can also control a soft microrobot (with two DOFs) by changing the direction of the magnetic field.

The diameter of the guidewire (Hi-Torque Powerturn hydrophilically coated guidewire; Abbott Vascular, Santa Clara, CA) connected to the microrobot is 0.36 mm, and its length is 190 cm. The guiding catheter (Cordis, Miami Lakes, FL) is a flexible thin plastic tube that is 1.67 mm in diameter.

For the *in vitro* experiments, a 2D blood vessel phantom was fabricated by a 3D printer (ProJet MJP 3500; 3D Systems, Rock Hill, SC) as shown in Figure 6 (top left). Both the width and the depth of the 2D phantom were 2 mm, and the branch angles varied from 30° to 120° to mimic the coronary arteries in a 2D environment. The 3D blood vessel phantom (Elastrat, Geneva, Switzerland) made of soft silicone mimics the aortic arch and the left and right coronary arteries, including the marginal and posterior intraventricular arteries of the latter artery. The left coronary artery includes the left anterior intraventricular and circumflex arteries.^41^

## Results

### Model analysis and feedforward angular steering

In the FEA simulation, the magnetic field directions, as shown in Figure 4, varied from 30° to 170° and the magnetic torque rotated the PDMS cantilever beam. Figure 4 shows the simulated (COMSOL) deformations of the soft microrobot caused by the external magnetic field. To have a condition similar to the model in the experiments, the microrobot was fixed at the end of a rigid holder. The experimental results with the guidewire and a rigid holder revealed negligible variation, 4% on average (Supplementary Fig. S1). The red arrows indicate the strength and direction of the magnetic field (the vector sums of the magnetic fields of the OctoMag and the two magnets). At regions distant from the two magnets, the field is influenced principally by the magnetic steering system (the external magnetic field) but, in regions close to the magnets, the field generated by those magnets is dominant. Initially, the magnets and the external field point in the same direction (Fig. 4a); however, structural limitations gradually render the magnets unable to fully follow the steering magnetic field. Figures 4a–f confirm that the flexible microrobot is deformed by the external magnetic field and that the deformation angle increases as the change from the original direction of the external magnetic field increases. Figure 4f shows that, at an external field direction of 170°, structural limitations render the microrobot unable to assume the same direction.

**FIG. 7.**
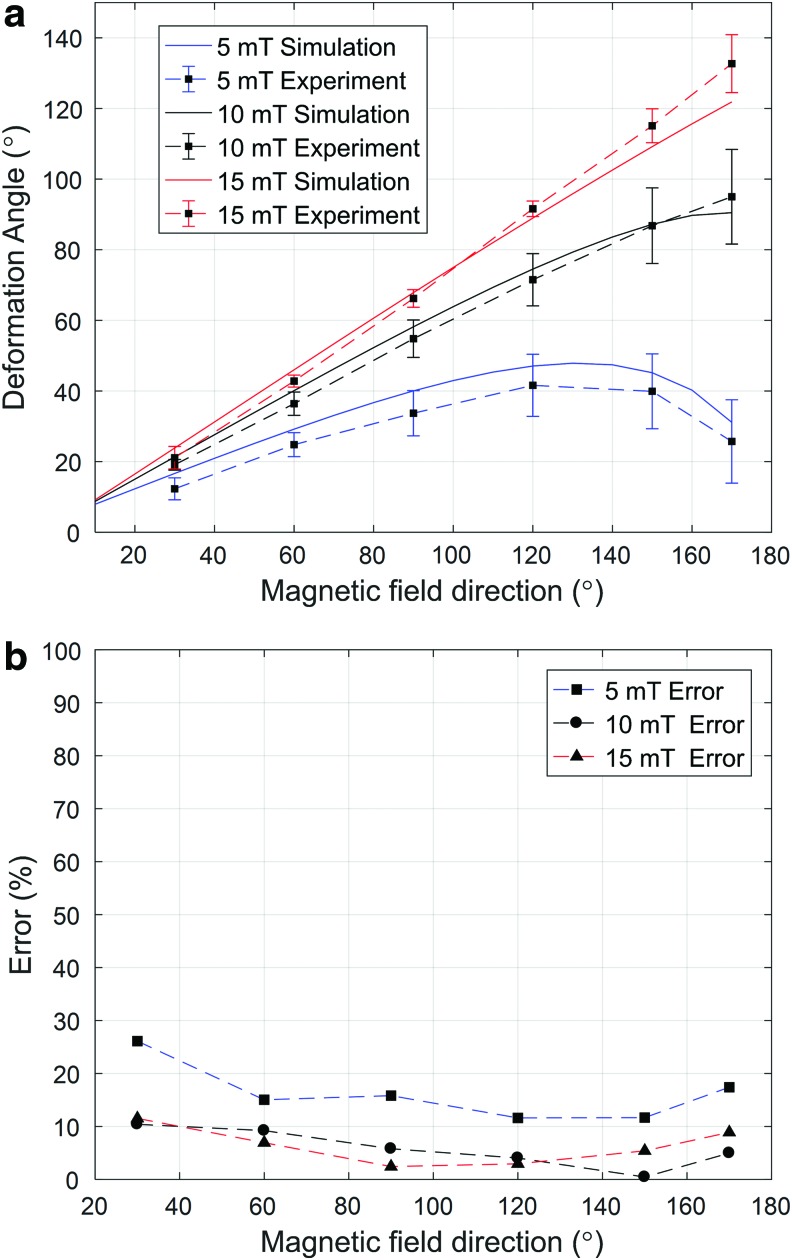
Simulated and experimental deformation angles when the field directions varied (*n* = 3 for the experimental results). **(a)** Simulation (field directions between 10° and 170°) and experimental (field directions 30°, 60°, 90°, 120°, 150°, and 170°) results. **(b)** The differences between the experimental results and the simulations for field directions of 30°, 60°, 90°, 120°, 150°, and 170°.

The measured and simulated deformation angles are compared in Figure 7a. The deformation angles change as the external magnetic field direction (${R_{roll}}$) varies from 30° to 170° at magnetic field intensities of 5, 10, and 15 mT. The maximum deformation angle yielded by the simulation was 121.8° when the direction of the external magnetic field (15 mT) was 170°; the measured, maximum, experimental deformation angle was 132.7° under these conditions.

Figure 7b shows the percentage differences between simulations and the experiments. All errors were <20%, showing that the simulations and experimental data agreed, with the exception of the results obtained by using a field of magnetic intensity of 5 mT at 30°.

### Soft microrobot trajectory tracking in the 2D phantom

The main function of the developed soft microrobot is to navigate a conventional guidewire in multi-blood-vessel branches. We tested trajectory tracking *in vitro* in a water-filled 2D phantom placed in the region of interest of the OctoMag. Microrobot rotation was controlled by changing the direction of the magnetic field, and rectilinear motion was controlled by the master/slave system. A trajectory planning algorithm was developed to enhance the human operator navigation capabilities. The trajectory information consists of the vessel length (rectilinear displacement) and the vessel angle in the bifurcations (rotational displacement) extracted and used to estimate rectilinear and rotational displacements.

**FIG. 8.**
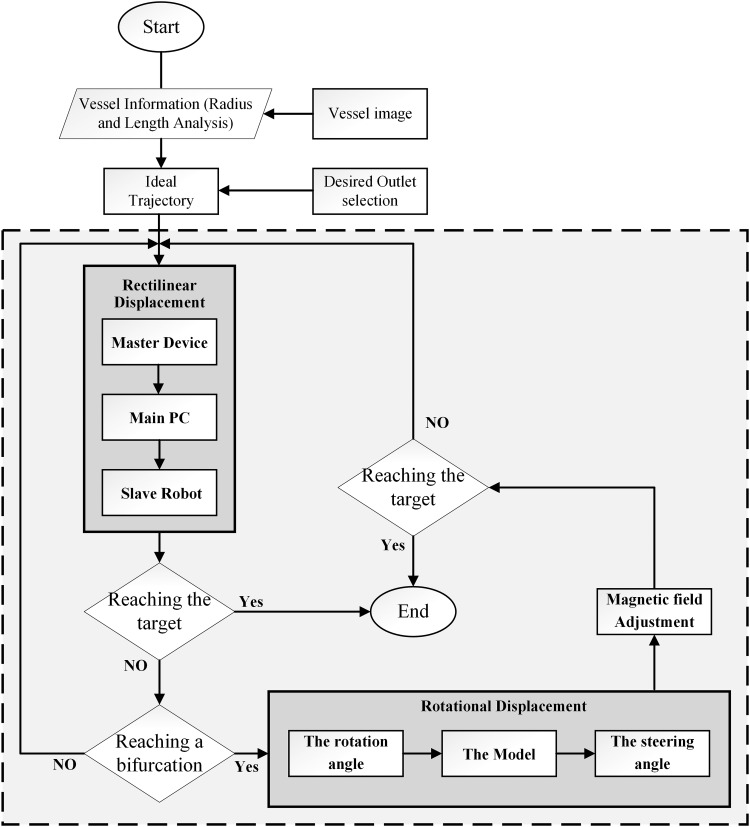
Trajectory planning flowchart for 2D tracking.

**FIG. 9.**
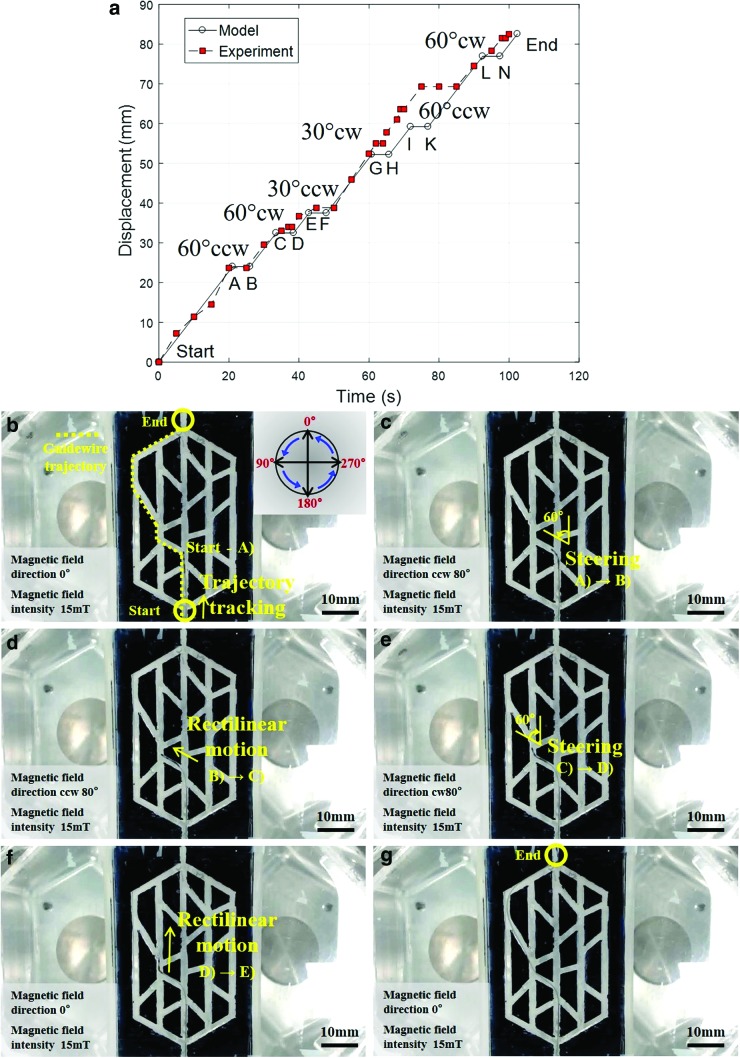
*In vitro* tracking within a 2D blood vessel phantom with various branches and inclinations. **(a)** The time-displacement trajectory for the desired path (the angles are the required deformation angles). **(b)–(f)** the steering and rectilinear motions of the soft microrobot and guidewire; and **(g)** attainment of the goal position. cw, clockwise; ccw, counterclockwise.

The delegated task of GSM navigation is a semi-automatic task. Modeling is used to predict the linear and rotational displacements, and an operator guarantees the needed high precision. The flowchart in Figure 8 shows the trajectory tracking process (the OctoMag, the master/slave system, and the optical camera). As the flowchart indicates, vessel data (length, diameter, and direction) were initially calculated by using the MATLAB software (MathWorks, Inc., Natick, MA). When a 20 mm/s push velocity was demanded by the master system, the scaling between the master and slave (200–11.4 mm) afforded a rectilinear velocity of 1.14 mm/s. The time required for rotational displacement was assumed to be 5 s. Based on this information, we generated the path shown in Figure 9a. This is a displacement-time diagram showing how to attain the target position using a predefined path. Rectilinear motion continues until the robot reaches the first bifurcation. Then, the direction of the magnetic field is changed based on the model developed in the previous section. Although the algorithm can predict linear and angular displacements with the developed models, the operator has complementary skills and can adjust the linear and angular displacements via master-slave and magnetic-field control systems. This process continues until the robot attains the desired position.

The guidewire trajectory is shown in Figure 9b (dotted line). The GSM is initially driven by the master/slave system. Once the GSM reaches a bifurcation (Fig. 9c) and must turn 60° counterclockwise (ccw), it is steered 80° ccw by the external magnetic field. Figure 9d shows the guidewire advancing along the planned trajectory and magnetic steering at the second branch (Fig. 9e; 60° clockwise [cw]). The GSM is controlled similarly in Figure 9f. Finally, the GSM attains the target (Fig. 9g). The Supplementary Video S1 shows the entire course of tracking inside the 2D phantom. The time-displacement diagram during 2D tracking is also shown in Figure 9a.

### Trajectory tracking in the 3D phantom

**FIG. 10.**
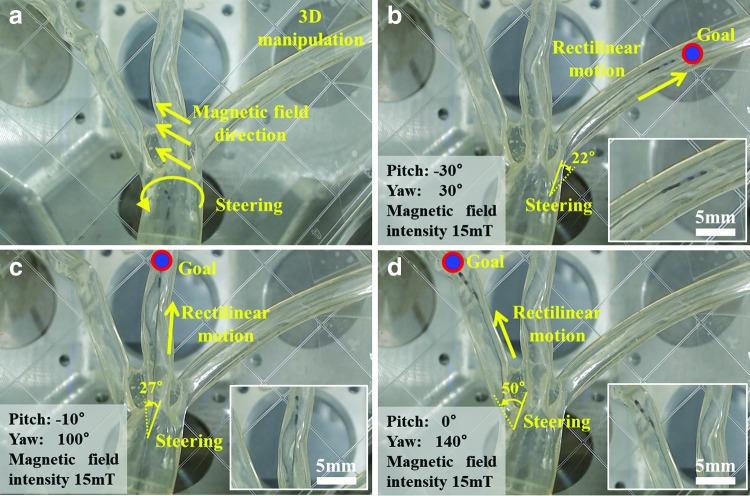
*In vitro* tracking within a 3D phantom of a left coronary artery with three branches. **(a)** 3D steering of the soft microrobot. **(b–d)** cw 22°, ccw 27°, and ccw 50° steering using the OctoMag to impart rectilinear motion via the master/slave system.

In the previous section, we used a 2D phantom. As the heart is a 3D structure, the microrobot must be controlled in 3D. Consequently, a transparent 3D phantom mimicking the coronary artery was used in a steering/tracking test within three branches of the left coronary artery (Fig. 10 and Supplementary Video S1). Given that the geometry of the 3D phantom was known, only the top camera was used for tracking. In future studies, a bi-plane X-ray system (two X-ray systems in a perpendicular orientation) will be used to register the patient's 3D CT image, enabling 3D locomotion in real time. The GSM is controlled by both the external magnetic field and the master/slave system.

The GSM was precisely steered, using the magnetic field (Fig. 10a), into each of the three branches as directed. To deliver the GSM into the right branch, deformations of −30° in pitch and 30° in yaw were required (Fig. 10b). When targeting the middle branch, the figures were −10° pitch and 100° yaw (Fig. 10c. When targeting the left branch, the figures were 0° pitch and 140° yaw (Fig. 10d). In conclusion, the microrobot exhibited dexterous 3D movement.

## Discussion

We expand next on our modeling and experimental findings:

1. Effects of magnetic intensity
2. Curvature of the deformation angle
3. Variations between the model and the experiments
4. Working conditions

### Effects of magnetic intensity

The change in deformation angle as the magnetic field changes is given by Equation (13). The deformation depends on the matrix *M* (representing magnet geometry), the matrix *β* (representing the mechanical properties of the microrobot), and the matrix *B* (representing magnetic field intensity and direction). As *θ_iyz_* and *θ_iy_*_x_ of matrix *M* change when the field direction changes, matrix *M* is a function of field direction. This explains the curvature of the deformation diagram (Fig. 7a); the magnitude of magnetization (*m*) is constant. The deformation varies, however, with changes in magnetic intensity (matrix *B*). A higher intensity increases curvature, which is associated with greater deformation.

For the field intensities of 5 and 10 mT, the maximum average deformation angles achieved in the experiments were 41.6° and 95°, respectively. To achieve higher steerability, the field intensity was set to 15 mT, associated with a maximum average deformation angle of 132.7° in the experiments.

### Curvature of the deformation angle

**FIG. 11.**
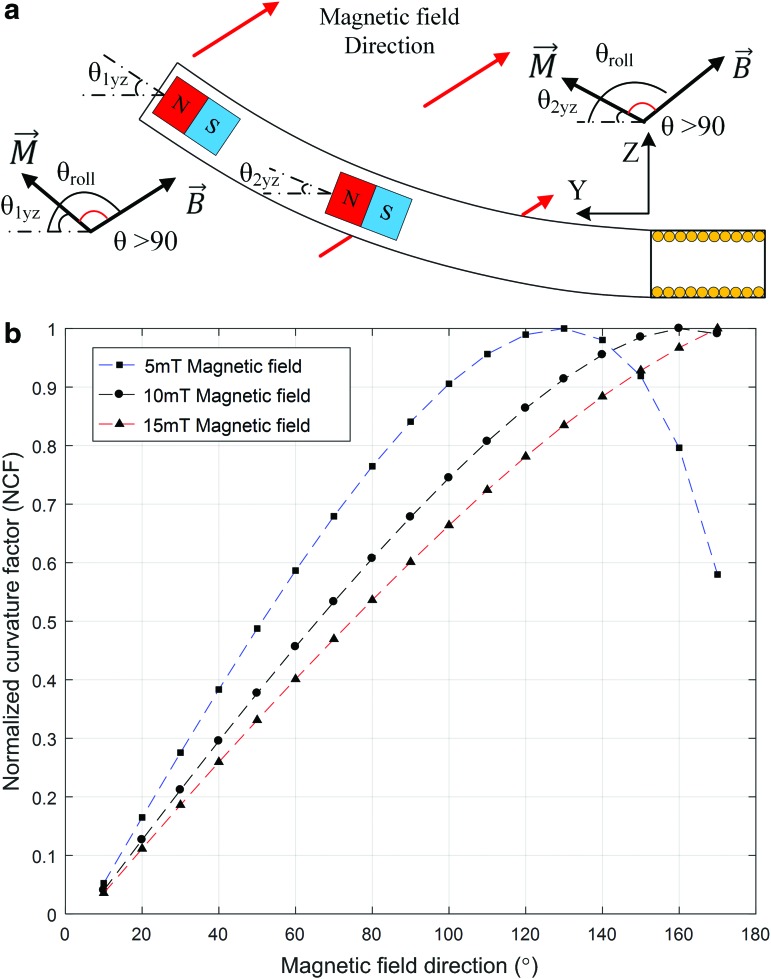
**(a)** Schematic of microrobot deformation by magnetic torque when θ > 90. **(b)** Simulation of the normalized curvature factor by variation in magnetic field direction.

The curvature of the deformation angle (both measured and simulated) was nonlinear (Fig. 7a). Initially, the microrobot deforms in a linear manner as the external field angle increases. However, once the field direction attains 130° (for a 5-mT field) or 160° (for a 10-mT field), continued changes in field direction reverse the effects of angular deformation. The extent of deformation [as shown in Eq. (13)] depends on the magnetic torque (the force is considered to be zero). The value of torque [Eq. (4)] depends on the external magnetic intensity ($\left\vert B \right\vert$), the magnetization vector ($\left\vert M \right\vert$), and the sine of the angle between the two vectors (*θ*) (schematic of Fig. 11a). As both the magnetic field intensity and the magnetization vector are constant, the torque declines when *θ* passes through 90°, reducing the deformation angle.

The difference in curvature is represented by Equation (14). In the *yz* plane, *i = 2, θ_yaw_ = 0,* and *β_1_ = β_2_ = β*; Equation (14) can then be simplified to:

\begin{align*}
CF = \left[ { \begin{matrix} 0 & { - ( s{ \theta _{1yz}} + s{ \theta _{2yz}} ) } & {c{ \theta _{1yz}} + c{ \theta _{2yz}}} \\ {s{ \theta _{1yz}} + s{ \theta _{2yz}}} & 0 & 0 \\ {c{ \theta _{1yz}} + c{ \theta _{2yz}}} & 0 & 0 \\ \end{matrix} } \right] diag{ ( m \beta B ) _{3 \times 3}} \left[ \begin{matrix} 0 \hfill \\ c{ \theta _{roll}} \hfill \\ s{ \theta _{roll}} \hfill \\\end{matrix}  \right] \tag{16}
\end{align*}

where *CF* is the curvature factor. The curvature factor *CF* depends on ${ \theta _{1yz}}$, ${ \theta _{2yz}}$, and *θ_roll_*. ${ \theta _{1yz}}$ and ${ \theta _{2yz}}$ are calculated by using the deformation angles of the model (Fig. 7a) and *θ_roll_* ranges between 10° and 170°. To eliminate the effects of *diag*(*mβB*), a normalized curvature factor (*NCF*) is derived [*NCF = CF/max(CF)*] (Fig. 11a).

The *NCF* for a field of intensity 5 mT declines at *θ_roll_* >130°, reducing the magnetic torque. At a field intensity of 10 mT, the *NCF* peaks at 160° and declines slightly thereafter. Finally, at a field intensity of 15 mT, the *NCF* does not peak, and instead increases continuously. Figure 11a, b explains the curvature shown in Figure 7a.

### Variation between the experimental data and simulations

With the exception of the 30° direction, the model followed the experimental results closely. For the 30° condition, the magnitude of the deviation between the experimental and simulation results for 10 and 15 mT was within the ranges of acceptable deviation (10% and 11%, respectively). For the 5-mT condition, the model and experimental curvatures were similar, and the magnitude of the deviation between the model and experiments changed only slightly (Fig. 7). Nevertheless, because the magnitude of the deformation in the model was low (Fig. 7) in error calculations, a maximum error of 26.1% was observed at 30°.

### Working conditions

A high steering angle was achievable in a magnetic field of 15 mT. Linear behavior was observed both experimentally and in simulations. To avoid possible microrobot buckling, we suggest that the magnetic field direction be held within 0–150°. The error is then 11.5%, rendering the results more reliable.

## Conclusion

We developed a soft microrobot improving intravascular guidewire steerability and a model for analysis of angular deformation. The microrobot, composed of PDMS, a microspring, and two permanent magnets, was fabricated by casting using a PDMS mold. Our mathematical model successfully estimated microrobot deformation; the experimental data and the simulations were in good agreement. The deformation angle depends on both the direction and intensity of the magnetic field. The intensity is held constant, and the field direction is used for steering. The experimental deformation angle ranged from 21.1° to 132.7° at a magnetic field intensity of 15 mT. Therefore, the soft microrobot could be steered inside a complex 2D phantom. To explore possible applications in terms of PCI, we performed *in vitro* steering and tracking tests inside a water-filled 2D phantom mimicking the coronary arteries. The microrobot engaged in rectilinear motion and traced a complex path controlled by the direction of the magnetic field. Similarly, a 3D phantom of the left coronary arteries was used to test 3D tracking performance. The microrobot was successfully guided into any desired arterial branch. Thus, our system improves guidewire steerability and will find applications in robot-assisted PCI procedures. The Supplementary Video S1 shows successful 2D and 3D tracking by the soft microrobot. The results presented in this work show a microrobotic approach for improving conventional guidewire navigation. In the future, the developed approach can be used with a conventional catheter for angioplasty intervention. It also can be used with catheters equipped with novel magnetically actuated devices. Further, because perforation is of great concern in angioplasty procedures, the shape of the soft microrobotic tip should be considered in future studies.

## Supplementary Material

Supplemental data

Supplemental data
