# Supplementary material for: A Magnetically Controlled Soft Microrobot Steering a Guidewire in a Three-Dimensional Phantom Vascular Network
Source: Soft Robot. 2019 Feb 11;6(1):54–68. doi: 10.1089/soro.2018.0019 (PMC6386781; doi:10.1089/soro.2018.0019)
Supplement: Supplemental data [file Supp_Data.docx]

**SUPPLEMENTARY TEXT**

A Magnetically Controlled Soft Microrobot Steering a Guidewire in a Three-Dimensional Phantom Vascular Network

**Sungwoong Jeon^1,2,+^, Ali Kafash Hoshiar^1,2,+^, Kangho Kim^1,2,+^, Seungmin Lee^1,2^, Eunhee Kim^1,2^, Sunkey Lee^1,2^, Jin-young Kim^1,2^, Bradley J. Nelson^2,3^, Hyo-Jeong Cha^4^, Byung-Ju Yi**^4^, **, Hongsoo Choi^1,2*^**

^1^Department of Robotics Engineering, Daegu Gyeongbuk Institute of Science and Technology (DGIST), Daegu, South Korea

^2^DGIST-ETH Microrobotics Research Center (DEMRC), DGIST, Daegu, South Korea

^3^Institute of Robotics and Intelligent Systems, ETH Zurich, CH-8092 Zurich, Switzerland

^4^Department of Electronic Systems Engineering, Hanyang University, Ansan, Korea

^+^These authors contributed equally to this work.

*E-mail: mems@dgist.ac.kr

Additional experiments were conducted with the microrobot fixed at the end of the guidewire. In the experiment, the guidewire length was 10 mm. Although there was some variation between experimental results in using a rigid holder only and a guidewire with a rigid holder, the difference was negligible (4% on average), as shown in Supplementary Fig. S1.

**(a)**

| 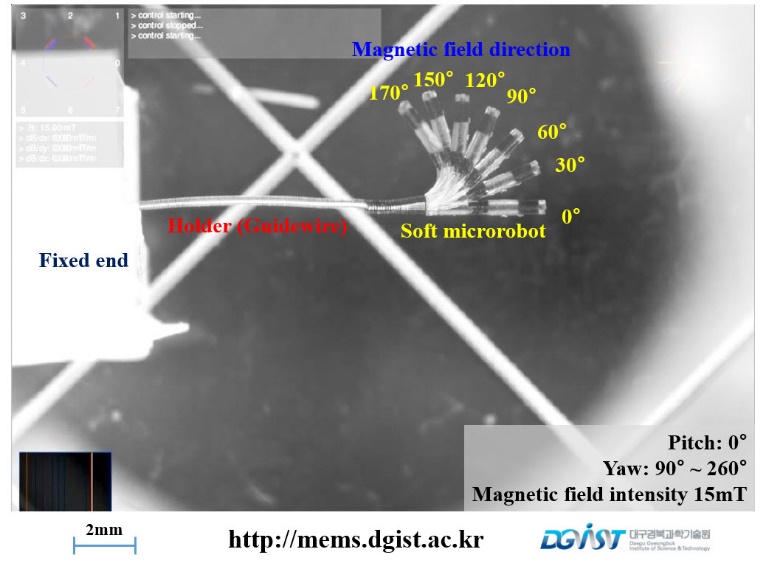  **(b)** |
| --- |
|   SUPPLEMENTARY FIG. S1. **(a)** Superimposed image of microrobot steering with a guidewire as a holder and **(b)** the deformation angles for the rigid holder and guidewire holder for the same microrobot. |

To verify the biocompatibility and cytotoxicity of the soft microrobot, human colorectal cancer (HCT116) cells were cultured with a soft microrobot (cut to 2 mm) and without a soft microrobot (control group) in a 24-well plate for 3 days (Fig. S1). The viability of the HCT116 cells cultured with the soft microrobot was checked after 3 days of cell culture using the LIVE/DEAD Cell Imaging Kit according to the manufacturer’s instructions (488/570, Molecular Probes, Life Technologies Corp., CA, USA). The live/dead cell viability assay uses a cell-permeable dye to stain live cells; a cell-impermeable dye was used to stain dead and dying cells, which are characterized by compromised cell membranes (Figs. S2 and S3). In live cells, the calcein assay uses the conversion of non-fluorescent calcein AM dye to fluorescent calcein dye through intracellular esterase activity after permeating live cells. The dead cell component is membrane-impermeant and does not enter viable cells with intact membranes. In dying and dead cells, which have damaged membranes, a bright red fluorescence is generated upon binding to nucleic acids. The figures show biocompatibility test results in the form of bright-field and fluorescence images of HCT116 cells with the soft microrobot. The green fluorescence signal, which accounts for most of the fluorescence image, and the absence of the red fluorescence signal, demonstrate very high cell viability and the biocompatibility of the soft microrobot (Fig. S2).


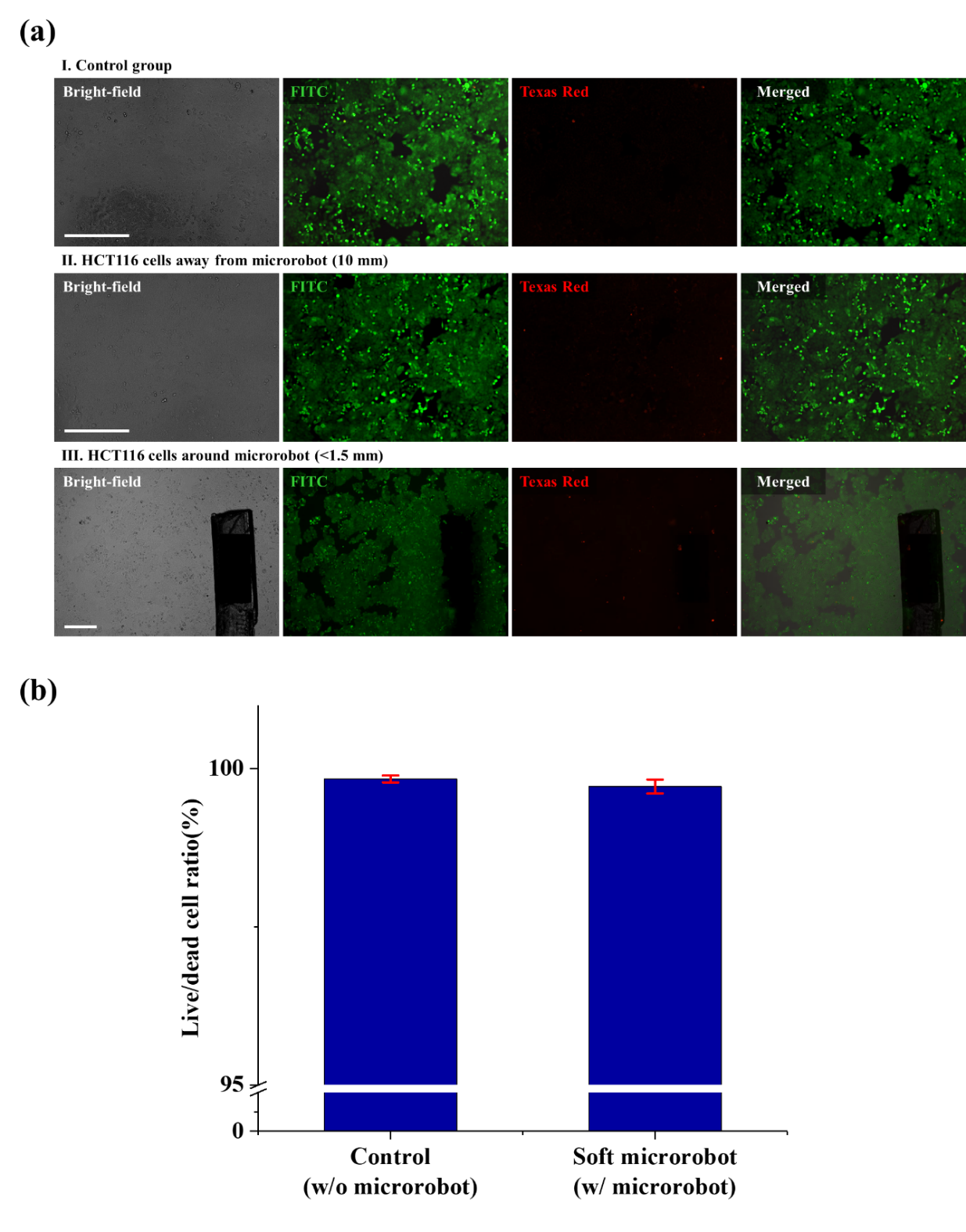


SUPPLEMENTARY FIG. S2. Cell viability assay of the soft microrobot. **(a)** Bright-field and fluorescent images of HCT116 cells cultured with the soft microrobot for 3 days (*green*: live cells; *red*: dead cells): I) cultured w/o the soft microrobot (control group), II) located 10 mm away from the soft microrobot, and III) located 1.5 mm around soft microrobot. **(b)** Cell viability relative to the control. (Scale bar is 400 µm.)

**References**

S1. Jiao J, Sun L, Guo Z, *et al*. Antibacterial and anticancer PDMS surface for mammalian cell growth using the Chinese herb extract paeonol (4-methoxy-2-hydroxyacetophenone). Sci Rep 2016;6:38973.

S2. Lee S, Lee S, Kim S, *et al*. Fabrication and characterization of a magnetic drilling actuator for navigation in a three-dimensional phantom vascular network. Sci Rep 2018;8:3691.

S3. Kim E, Yoo SJ, Kim E, *et al*. Nano-patterned SU-8 surface using nanosphere-lithography for enhanced neuronal cell growth. Nanotechnology 2016;27:175303.
